# Neonatal risk factors for respiratory morbidity during the first year of life among premature infants

**DOI:** 10.1590/S1516-31802006000200006

**Published:** 2006-03-02

**Authors:** Rosane Reis de Mello, Maria Virgínia Peixoto Dutra, José Roberto Ramos, Pedro Daltro, Márcia Boechat, José Maria de Andrade Lopes

**Keywords:** Risk factors, Respiratory tract diseases, Premature infant, Respiratory mechanics, Tomography, Fatores de risco, Doenças respiratórias, Prematuro, Mecânica respiratória, Tomografia

## Abstract

**CONTEXT AND OBJECTIVE:**

There have been dramatic increases in very low birth weight infant survival. However, respiratory morbidity remains problematic. The aim here was to verify associations between pulmonary mechanics, pulmonary structural abnormalities and respiratory morbidity during the first year of life.

**DESIGN AND SETTING:**

Prospective cohort study at Instituto Fernandes Figueira, Fundação Oswaldo Cruz, Rio de Janeiro.

**METHODS:**

Premature infants with birth weight < 1500 g were studied. Lung function tests and high-resolution chest tomography were performed before discharge. During the first year, infants were assessed for respiratory morbidity (obstructive airways, pneumonia or hospitalization). Neonatal lung tests and chest tomography and covariables potentially associated with respiratory morbidity were independently assessed using relative risk (RR). RR was subsequently adjusted via logistic regression.

**RESULTS:**

Ninety-seven newborn infants (mean birth weight: 1113g; mean gestational age: 28 weeks) were assessed. Lung compliance and lung resistance were abnormal in 40% and 59%. Tomography abnormalities were found in 72%; respiratory morbidity in 53%. Bivariate analysis showed respiratory morbidity associated with: mechanical ventilation, prolonged oxygen use (beyond 28 days), oxygen use at 36 weeks, respiratory distress syndrome, neonatal pneumonia and patent ductus arteriosus. Multivariate analysis gave RR 2.7 (confidence interval: 0.7-10.0) for simultaneous lung compliance and chest tomography abnormalities. Adjusted RR for neonatal pneumonia and mechanical ventilation were greater.

**CONCLUSIONS:**

Upon discharge, there were high rates of lung mechanism and tomography abnormalities. More than 50% presented respiratory morbidity during the first year. Neonatal pneumonia and mechanical ventilation use were statistically significant risk factors.

## RESUMO

**CONTEXTO E OBJETIVO:**

Na última década ocorreu aumento dramático na sobrevida dos prematuros de muito baixo peso ao nascer. Contudo, a morbidade respiratória é ainda um problema. O objetivo deste estudo é verificar a associação entre mecânica pulmonar, avaliação estrutural pulmonar e a morbidade respiratória no primeiro ano de vida.

**TIPO DE ESTUDO E LOCAL:**

Coorte prospectivo realizado no Instituto Fernandes Figueira, Fundação Oswaldo Cruz, Rio de Janeiro, Brasil.

**MÉTODOS:**

Participantes: prematuros com peso de nascimento inferior a 1.500 g. Antes da alta hospitalar foram realizadas prova de função pulmonar e tomografia computadorizada de tórax de alta resolução. As crianças foram acompanhadas durante o primeiro ano de vida e avaliadas quanto à presença de morbidade respiratória (síndrome obstrutiva de vias aéreas, pneumonia, internação). Resultados da prova de função pulmonar, tomografia de tórax e co-variáveis potencialmente associadas com morbidade respiratória foram avaliadas independentemente, usando-se risco relativo (RR). Os riscos relativos foram posteriomente ajustados através de regressão logística.

**RESULTADO:**

Estudamos 97 prematuros com peso de nascimento médio de 1.113 g e idade gestacional 28 semanas. A complacência pulmonar estava alterada em 40% dos casos e a resistência, em 59%. Alterações tomográficas ocorreram em crianças, morbidade respiratória em 53%. Na análise bivariada, as co-variáveis associadas com a morbidade respiratória foram: ventilação mecânica, uso prolongado de oxigênio (além de 28 dias), oxigênio às 36 semanas, doença de membrana hialina, pneumonia neonatal, persistência do canal arterial. Na análise multivariada o risco para alteração simultânea da complacência pulmonar e tomografia de tórax foi 2,7 (intervalo de confiança, IC: 0,7-10,0). No cálculo do risco ajustado as co-variáveis pneumonia neonatal e ventilação mecânica obtiveram uma magnitude maior.

**CONCLUSÃO:**

Encontramos altas taxas de alterações na mecânica pulmonar e na tomografia de tórax. Mais de 50% das crianças apresentaram morbidade respiratória no primeiro ano de vida. Os fatores de risco estatisticamente significativos foram pneumonia neonatal e assistência ventilatória.

## INTRODUCTION

Because of lung immaturity, premature infants are exposed to factors that are potentially harmful to their lungs. Premature infants present respiratory distress syndrome, apnea and infections that usually lead to the use of oxygen and mechanical ventilation. This may cause a higher risk of pulmonary injury. Several perinatal factors may trigger a sequence of events that lead to persistent structural pulmonary abnormalities and consequent increased incidence of respiratory morbidity in the future. Some authors have shown an association between prolonged use of oxygen, use of mechanical ventilation during the neonatal period and respiratory symptoms during infancy among premature children.^1^ In one study,^2^ around 30% of the infants with birth weight less than 1500 g presented respiratory symptoms during their first year of life. Those that remained symptomatic during the second year were ventilated for longer periods and presented higher rates of abnormal pulmonary function test at six months of age than did those who were only symptomatic during the first year of life.

Respiratory diseases are the main causes of hospitalization for such infants, with particular emphasis on pneumonia and episodes of wheezing.^3^ Very low birth weight premature infants present a significantly greater risk of wheezing and hospitalization^4^ than do children with birth weight greater than 2500 g. The variables presenting the greatest risk in relation to wheezing were male gender and bronchopulmonary dysplasia, while prolonged oxygen use and wheezing were risk factors for hospitalization.^5^

Mechanical ventilation, even using low pressure and tidal volume can damage the lungs and affect the respiratory function of premature infants during their follow-up.^2^ According to Martinez et al. (1991),^6^ abnormalities in pulmonary function precede and predict the development of respiratory disease with wheezing during the first year of life. However, few studies have evaluated the association between abnormal pulmonary function or neonatal pulmonary structure and future respiratory problems.

## OBJECTIVE

The objective of the present study was to verify the possible associations between functional and structural pulmonary abnormalities during the neonatal period and respiratory morbidity during the first year of life, among premature infants.

## METHODS

This was a prospective cohort study. The study sample included premature infants with birth weight (BW) lower than 1500 g and gestational age (GA) less than 34 weeks. All these infants were born at Instituto Fernandes Figueira, a tertiary maternal and child care hospital in Rio de Janeiro, Brazil, between January 1998 and August 2000. They were followed up at the hospital's high-risk infant follow-up outpatient clinic, until they reached the corrected age of 12 months. Small for gestational age (SGA) newborns^7^ and infants with genetic syndromes, malformations and congenital infections were excluded from the study.

Informed consent was obtained from a parent or caregiver, for the child to participate in the tests. This project was approved by the Ethics Committee of Instituto Fernandes Figueira.

### Procedures and main measurements

#### Evaluation of pulmonary function

Pulmonary function tests were performed during the week scheduled for hospital discharge, with the child clinically stable and not using oxygen therapy. Pulmonary resistance and compliance were verified. The tests were performed by a physician who was unaware of the infant's clinical history. They were performed with the infant inside an incubator, during non-rapid eye movement sleep, without sedation.

The evaluation of pulmonary function included measurement of the physiological signs of flow, esophageal pressure and airway pressure. Airflow in the airways was measured using a resuscitation-type mask coupled to a pneumotachograph (Fleisch 00) and an ultrasensitive pressure transducer (Validyne MP45). Tidal volume was obtained through electronic integration of the recorded flow signal. Esophageal pressure was measured using a water-filled catheter positioned in the distal third of the esophagus. Airway pressure was measured using a catheter coupled to the resuscitation mask, connected to a Validyne DP 45 pressure transducer.

The data collected were keyed into a microcomputer. The acquisition software for analog signs was PCLAB (Data Translations). The digitized data were analyzed and processed using the Anadat/Labdat software (Infodat). Between 20 and 50 respiratory cycles per recorded file were selected for calculating pulmonary resistance and compliance, using linear regression (computed calculation; Abreath-Anadat). Pulmonary compliance values were calculated and corrected according to body weight.

Abnormal pulmonary compliance was defined as less than 1.2 ml/cmH_2_O/kg.^8^ Abnormal pulmonary resistance was defined as greater than 50 cmH_2_O/l/second.^9^

#### Pulmonary structural evaluation

Thoracic high-resolution computed tomography (HRCT) was performed during the week scheduled for hospital discharge, preferably during physiological sleep and without sedation.

The equipment used was spiral computed tomography (CT) (General Electric, model ProSpeed S), using high-resolution slices of 90 mA (60 mA for 1.5 sec). The technique utilized was as recommended by Lucaya et al. (1996),^10^ utilizing low-dose radiation. Six to nine axial slices were used, with one-millimeter thickness and 10 to 15 millimeter intervals.

The films were reviewed independently by two radiologists without knowledge of the infant's history, and inter-observer agreement was verified. Cases of discordant inter-observer results were resolved by seeking a consensus between the specialists.

Abnormal test results were defined as those displaying one or more of the following characteristics: aeration disorder, parenchymatous bands, atelectasis, parenchymatous consolidation, ground-glass images, thickening of the bronchial wall, thickening of the interlobular septum, sub-pleural opacity, or bubbles/cysts.^11^

For analytical purposes, the CT results were grouped according to the number of abnormalities: zero (normal test); one or two; and three or more.

Data on the newborn infants were recorded prospectively and included birth weight, gestational age, Clinical Risk Index for Babies (CRIB),^12^ utilization of mechanical ventilation, utilization of oxygen therapy and concomitant clinical events during hospitalization. Data were also recorded regarding the mother and/or father's smoking habits, the number of children less than five years of age in the household, and breastfeeding.

Bronchopulmonary dysplasia was considered to be due to dependence on oxygen therapy beyond the corrected age of 36 weeks. Oxygen use was considered to be "prolonged" if the baby required oxygen therapy after reaching 28 days of life.

#### Clinical evaluation during the first year of life

The infants were monitored by a pediatrician, monthly in the outpatient follow-up clinic or according to clinical need. Each visit included a physical examination, and data were collected from the infant's parent or caregiver regarding clinical events since the previous visit.

Respiratory morbidity was defined as the presence of one or more of the following:

- *Obstructive airway syndrome*: presence of two or more episodes of wheezing causing respiratory difficulty,^13^ observed by the pediatrician by means of pulmonary auscultation, and which which required bronchodilatory medication.
- *Hospitalization due to respiratory problems:* hospitalization of the infant for more than 24 hours.^14^
- *Pneumonia*: presence of tachypnea, inter-and sub-costal retraction, crackles and proven radiological abnormalities.^2,14^ Chest X-rays were interpreted by a pediatric radiologist.

#### Statistical methods

The database creation and preliminary analyses used Epi 2000.^15^ Logistic regression used the Statistical Package for Social Science (SPSS) 8.0 for Windows.^16^

The main characteristics of the study sample were described by frequency measurements, and also means, medians and standard deviations. Statistical tests were used for differences in proportions (chi-squared). Statistical significance was set at 5%.

Incidence rates were calculated for single intercurrent respiratory events and respiratory morbidity (the occurrence of at least one of the intercurrent respiratory events) during the first year of life.

The neonatal pulmonary function test and high resolution CT results and all the covariables that were considered potentially associated with the outcome (respiratory morbidity) were analyzed individually and expressed as to relative risk (RR). This stage was followed by multivariate analysis (logistic regression).

## RESULTS

The initial study sample included 179 newborns with birth weight less than 1500 g and gestational age less than 34 weeks. Of these, 20 (11.2%) died during hospitalization, and four caregivers declined to authorize inclusion of their infants. A total of 58 neonates were excluded for other reasons (41 small for gestational age, three with congenital infection and 14 with genetic syndromes/malformations). Our final study sample thus consisted of 97 infants.

Around 32% of the mothers presented arterial hypertension during their pregnancy. Some 60% of the mothers had received antenatal steroid therapy. Cesarean delivery occurred in 51.5% of the newborn infants. Premature rupture of the chorioamniotic membranes was identified in 33 (34%) patients.

The sample characteristics are shown in Table 1, and intercurrent clinical events during the neonatal period are shown in Table 2.

**Table 1:** Characteristics of very low birth weight newborns with gestational age less than 34 weeks that were adequate for gestational age and born between 1998 and 2000 (n = 97) in Instituto Fernandes Figueira

Study sample characteristics	
Birth weight (g): mean ± SD	1113 ± 232.9
Gestational age (weeks): mean ± SD	28.5 ± 2.3
Male gender: n (%)	47 (48.5)
Newborn with birth weight < 1000 g: n (%)	28 (28.9)
Newborn with gestational age < 28 weeks: n (%)	42 (43.3)

*SD = standard deviation.*

**Table 2:** Intercurrent clinical events during the neonatal hospitalization period among 97 premature infants with birth weight less than 1500 g and gestational age less than 34 weeks that were adequate for gestational age and born between 1998 and 2000 in Instituto Fernandes Figueira

Intercurrent clinical events	n (%)
Patent ductus arteriosus	21 (21.6)
Apnea	65 (67.0)
Respiratory distress syndrome	45 (46.4)
Septicemia	64 (66.0)
Pneumonia	21 (21.6)

Slightly more than 30% of the cases were treated with exogenous surfactant (Table 3). The median duration of the use of mechanical ventilation was three days, and the mean duration was 12 ± 6 days. Considering only the infants who required mechanical ventilation, 52.2% (44 patients) still required oxygen therapy at 28 days of life and 22.7% (10 patients) at 36 weeks of corrected age. During the neonatal hospitalization period the mean duration of oxygen use was 24.7 ± 30.5 days.

**Table 3:** Therapeutic interventions during the neonatal hospitalization period among 97 premature infants with birth weight less than 1500 g and gestational age less than 34 weeks that were adequate for gestational age and born between 1998 and 2000 in Instituto Fernandes Figueira

Therapeutic interventions	n (%)
Use of mechanical ventilation	44 (45.4)
Use of surfactant	32 (33.0)
Use of oxygen for more than 28 days	24 (24.7)
Use of oxygen at 36 weeks (corrected for prematurity)	10 (10.3)
Use of oxygen for 10 days or more	36 (37.1)

At the time when the pulmonary function test was performed, the infants' mean weight was 1833 ± 426 g and their gestational age corrected for prematurity was 36 ± 2.4 weeks. The mean pulmonary compliance was 1.40 ± 0.47 ml/cmH_2_O/kg and mean pulmonary resistance was 60.0 ± 25.6 cm H_2_O/l/sec.

Among all the pulmonary function tests performed (n = 97), 35 (36.0%) presented normal compliance and resistance. In 39 cases (40.2%), compliance was abnormal (less than 1.2 ml/cmH_2_O/kg), and in 57 cases (58.8%), resistance was abnormal (greater than 50 cm- H_2_O/ml/sec). In 28 cases (28.9%), the pulmonary compliance or resistance was abnormal. Both pulmonary compliance and resistance were abnormal in 34 cases (35.0%).

### Thoracic high-resolution computed tomography

In the initial months of the study, 11 infants did not undergo thoracic CT, and thus the results from the analysis of thoracic high-resolution computed tomography were based on 86 infants.

The intra and inter-observer reliability in relation to normal and abnormal reports were investigated using the kappa coefficient.^17^ The intra-observer kappa coefficient for the two observers was 0.79. The kappa coefficient for inter-observer reliability for the two radiologists was 0.71.

Among the 86 high-resolution CTs performed, 24 (27.9%) were considered normal. The CT abnormalities are shown in Table 4: 32.6% (28) of them showed one or two abnormalities, while 39.5% (34) had three or more (Table 5).

**Table 4:** Abnormalities in thoracic highresolution computed tomography (HRCT) performed before hospital discharge of 86 premature infants with birth weight less than 1500 g born in Instituto Fernandes Figueira

HRCT abnormalities	n (%)
Aeration disorders	33 (38.4)
Parenchymal bands	30 (34.9)
Atelectasis	27 (31.4)
Ground-glass opacity	37 (43.0)
Thickening of the interlobular septum	16 (18.6)
Subpleural opacity	18 (20.9)
Consolidation	6 (7.0)
Bubble/cysts	8 (9.3)

**Table 5:** Number of abnormalities in thoracic high-resolution computed tomography (HRCT) performed before hospital discharge of 86 preterm infants with very low birth weight born in Instituto Fernandes Figueira between 1998 and 2000

Number of HRCT abnormalities	Number of patients n (%)
None	24 (27.9)
1 or 2	28 (32.6)
3 or more	34 (39.5)

Among the 22 children exposed to high oxygen concentrations (greater than 80%) during the neonatal period, 14 (64%) presented ground-glass images on CT, while of the 19 children that did not receive oxygen, only four (21%) presented ground-glass images (p = 0.006). Eight cases (36.4%) showed thickening of the interlobular septum on CT, out of the 22 exposed to high oxygen concentrations, whereas there was only one such case (5.2%) among the children that did not receive oxygen. The difference between these proportions was statistically significant. Consolidation was observed in 18% of the children exposed to high concentrations, while no cases of consolidation were observed among those that did not receive oxygen. The difference between these proportions was not statistically significant.

Initially, we proceeded with the analysis by verifying possible associations between covariables such as perinatal/neonatal antecedents and abnormalities seen via pulmonary function tests and high-resolution CT. The perinatal antecedents (delivery type, use of antenatal steroid therapy and arterial hypertension) did not show any association with lung mechanics (pulmonary compliance and pulmonary resistance), and not even by means of high-resolution computed tomography. Premature rupture of the chorioamniotic membranes was associated with chest tomography abnormalities (three or more abnormalities) (RR: 1.7; confidence interval, CI: 1.01-2.84).

Among the neonatal covariables, CRIB greater than four points (indicating more serious illness during the first hours of life) presented RR of 1.6 (CI: 1.03-2.5) for pulmonary compliance abnormalities, but without association with pulmonary resistance. It also presented an association with three or more tomography abnormalities (RR: 2.46; CI: 1.5-4.03). The covariables of respiratory distress syndrome and apnea did not show any significant risk with regard to pulmonary mechanics and chest tomography abnormalities.

Although children with patent ductus arteriosus have 60% higher risk of pulmonary compliance abnormalities, the confidence interval included the value one. The presence of patent ductus arteriosus showed an association with three or more tomography abnormalities (RR: 2.18; CI: 1.37-3.48).

The use of mechanical ventilation and continuous positive airway pressure (CPAP) presented significant risk in relation to tomography abnormalities, and the relative risks were respectively: 3.35 (CI: 1.78-6.3) and 2.65 (CI: 1.4-5.0).

Neonatal pneumonia presented relative risk of 2.02 (CI: 1.3-3.15) in relation to compliance value abnormalities and 2.47 (CI: 1.56-3.9) in relation to tomography abnormalities, but without an association with pulmonary resistance. Septicemia showed an association with pulmonary compliance (RR: 2.36; CI: 1.17-4.76) but not with pulmonary resistance and tomography abnormalities. Prolonged use of oxygen (after reaching 28 days of life) presented associations with abnormal pulmonary compliance (RR: 2.35; CI: 1.52-3.63), abnormal pulmonary resistance (RR: 1.52; CI: 1.13-2.05) and tomography abnormalities (RR: 3.27; CI: 2.01-5.32). Bronchopulmonary dysplasia (oxygen therapy at 36 weeks) presented associations with abnormal pulmonary compliance (RR: 2.61; CI: 1.83-3.73), abnormal pulmonary resistance (RR: 1.63; CI: 1.23-2.16) and tomography abnormalities (RR: 3.17; CI: 2.28-4.40).

### Respiratory morbidity during the first year of life

One child died during the study period due to pneumonia and septicemia. In 34% of the sample, one or both parents smoked. In 48.4% of the families, there were one or more siblings under five years of age in the household. In 44% of the families, there were three or more adults living in the household. Some 60% of the children were breastfed during the first year of life (n = 61).

We followed up all the children during their first year of life, and there were thus no losses to follow-up.

Among the 97 children, respiratory morbidity occurred in 52 (53%) cases. Signs of obstructive airway syndrome occurred in 27 (27.8%) of the children, pneumonia in 35 (36.1%) and hospitalization in 25 (25.8%).

Bivariate analysis was used for measuring the relative risk between the pulmonary function results, CT and clinical covariables, in relation to single intercurrent respiratory events and respiratory morbidity during the first year of life.

The following variables and covariables were associated with obstructive syndrome: abnormal pulmonary compliance, HRCT with three or more abnormalities, use of assisted ventilation, prolonged use of oxygen, neonatal pneumonia, use of oxygen for 10 days or longer, and patent ductus arteriosus.

The variables associated with pneumonia were: HRCT with three or more abnormalities, use of assisted ventilation, prolonged use of oxygen, use of oxygen for 10 days or longer, birth weight less than 1000 g, and mechanical ventilation for more than five days.

The variables associated with hospitalization were: use of assisted ventilation, prolonged use of oxygen, apnea, neonatal pneumonia, birth weight less than 1000 g, and mechanical ventilation for more than five days.

The independent variables associated with respiratory morbidity are shown in Table 6. The relative risk of the covariable "no breast-feeding" was 2.0 (CI: 1.0-4.1).

**Table 6:** Relative risk of respiratory morbidity during the first year of life among 97 very low birth weight premature infants, according to pulmonary function test, thoracic high-resolution computed tomography and neonatal intercurrent clinical events and therapeutic interventions

Exposure factors for respiratory morbidity	relative risk	confidence interval (95%)
Abnormal pulmonary compliance	1.49	1.04-2.13
Abnormal pulmonary resistance	1.33	0.89-1.99
HRCT: any abnormality	1.83	1.01-3.35
HRCT: 3 or more abnormalities	1.72	1.21-2.93
Mechanical ventilation	1.92	1.30-2.84
Prolonged use of oxygen (O_2_ > 28 days)	1.90	1.39-2.60
Oxygen use at 36 weeks (BPD)	1.59	1.09-2.35
Apnea	1.21	0.79-1.86
Respiratory distress syndrome	1.70	1.16-2.50
Neonatal pneumonia	1.91	1.41-2.60
Oxygen use ≥ 10 days	2.05	1.32-3.20
Patent ductus arteriosus	1.61	1.15-2.25
Mechanical ventilation ≥ 5 days	1.51	1.06-2.15
Gestational age < 28 weeks	1.31	0.90-1.89
Birth weight < 1,000 g	1.30	0.90-1.88
Septicemia	1.06	0.71-1.58
CRIB5	1.86	1.33-2.59
No breastfeeding	2.0	1.00-4.10

*HRCT = thoracic high-resolution computed tomography; CRIB5 = Clinical Risk Index for Babies with total score of more than four points; BPD = bronchopulmonary dysplasia.*

Multivariate analysis was done after concluding the bivariate analysis, and respiratory morbidity was the dependent variable. The independent variables tested in the multivariate model were those that presented statistical significance in the bivariate analysis. The logistic regression models were tested, and the model that was most adequate from both the statistical and biological points of view was selected.

Table 7 shows the adjusted relative risks, respective confidence intervals and information on the goodness-of-fit of the models, with respiratory morbidity as the dependent variable. We observed that the variables "neonatal pneumonia" and "assisted ventilation" presented a high risk of respiratory morbidity in all the models.

**Table 7:** Adjusted relative risk, respective confidence intervals (95%) and data on goodness-of-fit, according to various models for the outcome "respiratory morbidity"

	RR	Confidence interval (95%)			Model A (expanded)			
Variables included in model	Adjusted	Upper	Lower	p value	%cv Total	%cv No	%cv Yes	SigHL
Abnormal compliance	1.32	0.43	4.06	0.62	71%	69%	73%	0.39
CT: 3 or more abnormalities.	1.24	0.27	5.70	0.79				
Abnormal CT and compliance	2.06	0.26	16.03	0.49				
Prolonged use of oxygen (> 28 days)	0.83	0.11	6.02	0.85				
Neonatal pneumonia	2.94	0.66	13.15	0.16				
Patient ductus arteriosus	1.04	0.23	4.82	0.96				
Respiratory distress syndrome	1.80	0.58	5.64	0.31				
Mechanical ventilation	1.19	0.28	5.08	0.82				
Use of oxygen	1.21	0.26	5.73	0.81				
Length of hospitalization	1.01	0.98	1.04	0.67				
CRIB5	1.83	0.43	7.76	0.41				
Neonatal pneumonia	5.15	1.34	19.87	0.02	**Model B (stepwise)**			
Mechanical ventilation	3.29	1.33	8.16	0.01	%cv Total	%cv No	%cv Yes	SigHL
					68%	67%	69%	0.68
CT: 3 or more abnormalities	1.82	0.64	5.18	0.26	**Model C (Epidemiological)** (*)			
Neonatal pneumonia	4.37	1.11	17.36	0.04	%cv Total	%cv No	%cv Yes	SigHL
Mechanical ventilation	2.73	1.04	7.17	0.04	68%	67%	69%	0.81
Abnormal compliance	1.82	0.72	4.63	0.21	**Model D (Epidemiological)** (*)			
Neonatal pneumonia	4.49	1.14	17.81	0.03	%cv Total	%cv No	%cv Yes	SigHL
Mechanical ventilation	3.29	1.32	8.22	0.01	68%	67%	69%	0.40
Abnormal CT and compliance	2.68	0.72	10.07	0.14	**Model E (Epidemiological)** (*)			
Neonatal pneumonia	5.15	1.34	19.9	0.06	%cv Total	%cv No	%cv Yes	SigHL
Mechanical ventilation	3.29	1.33	8.16	0.05	70%	71%	69%	0.58

*RR = relative risk; CT = computed tomography; CRIB5 = total score of more than four points in the Clinical Risk Index for Babies; %cv = percent correct values; SigHL = signifance in likelihood ratio test (Hosmer Lemeshow test).*

(*) *Epidemiological method: stepwise forward method including variables with major epidemiological significance, albeit without statistical significance.*

We observed similar goodness-of-fit in the epidemiological models C, D and E, in relation to the percentages of correct values and the significance in the likelihood ratio test (Hosmer-Lemeshow test).^18^ In model E, although the variables of abnormal pulmonary compliance and abnormal CT had adjusted relative risks of close to three for respiratory morbidity, they were not statistically significant because the confidence interval ranged from 0.72 to 10.07.

## DISCUSSION

This is one of the few studies in Brazil presenting follow-up on a cohort of adequate for gestational age (AGA) very low birth weight newborns in relation to respiratory morbidity. One of the highly important features of this study was the absence of losses over 12 months of follow-up among the 97 children, who came from a low-income population. It is also a pioneering study in its use of thoracic high-resolution computed tomography during the neonatal period among premature infants.^19^ It is one of the few Brazilian studies to evaluate pulmonary function in a cohort of all-adequate for gestational age very low birth weight newborns.^19^

Premature birth is associated with interruption of the normal pattern of pulmonary development, which may result in abnormalities of lung mechanics and airway properties. Pulmonary function abnormality at an early age would explain the high morbidity-mortality among these children due to respiratory diseases during their first year of life.^20,21^ Our results appear to confirm those reported by the above-cited authors. Abnormal pulmonary compliance was a risk factor for wheezing. Although pulmonary resistance showed a relative risk of two in the bivariate analysis, this risk did not present a statistically significant confidence interval (CI: 0.94-4.29) for obstructive syndrome. Giffin et al. (1994)^22^ also found no significant association between raised pulmonary resistance and obstructive syndrome during the first year of life. However, increased resistance at 12 months represented an increased risk of respiratory morbidity at two years (RR: 2.89; CI: 1.84-4.5).

Martinez et al. (1988)^23^ reported that children with wheezing during their first year of life had significantly lower levels of respiratory conductance (the inverse of resistance) prior to developing their disease. Respiratory conductance depends on conductance in the lower and upper airways, lung tissue and chest wall. However, there may be differences in the caliber or length of the airways, as well as in lung and chest wall elasticity, comparing children with wheezing in the presence of respiratory tract viral disease and those without wheezing due to similar disease. These observations agree with our results, in which low pulmonary compliance (reflecting abnormal pulmonary elasticity) and high resistance, which can result from decreased airway caliber, were related to raised risk of obstructive syndrome.

We observed that 72% of the CT results showed abnormalities, with several concomitant abnormalities in 39%. Several authors have attempted to correlate CT and histopathological abnormalities. These studies were conducted mainly on animal models or among adults with respiratory disease. Ichikado et al. (2000)^24^ conducted a study on piglets six to nine weeks old that were subjected to high concentrations of oxygen (more than 80%). They presented areas with increased attenuation and thickening of the interlobular septum, as seen on thoracic CT. The CT and histopathological findings presented a correlation.

Among the 22 children in our study who received an inspired oxygen fraction greater than 80%, in 64% of the cases the chest CT showed areas with increased attenuation (ground-glass image). In comparison, among the children who did not receive oxygen, areas with increased attenuation occurred in only 21%. The difference between these proportions was statistically significant, as was the comparison between the children who received an inspired oxygen fraction greater than 80% and those who did not receive O_2_, in relation to thickening of the septum.

When the images were grouped according to the number of CT abnormalities, the presence of three or more abnormalities was statistically significant in the bivariate analysis, in relation to obstructive syndrome and pneumonia, but not to hospitalization. Yuksel et al. (1991)^25^ investigated the risk of the presence of radiographic abnormalities in relation to respiratory morbidity and hospitalization. These authors evaluated chest X-rays performed at one month of age on premature infants with a mean gestational age of 27 weeks. The X-rays were scored according to the presence of opacification, interstitial abnormalities and cysts. This radiological evaluation showed sensitivity of 88% for the detection of children with severe pulmonary functional abnormalities at six months of age. The radiographic abnormalities were associated with late indicators of respiratory complications in very low birth weight premature infants (use of bronchodilators and/or corticoids during the first two years; hospitalization; and diagnosing of asthma after reaching two years). The results indicated that the radiological abnormalities were more predictive of long-term respiratory complications than other commonly used criteria such as oxygen therapy at 28 days and 36 weeks, and diagnosis of bronchopulmonary dysplasia.^26^

Thomas et al. (2003)^27^ reported that chest X-ray abnormalities at 36 weeks of age (corrected for prematurity) were predictive of wheezing at six months of life. There are reports of increased risk of wheezing among children with siblings in the household or who shared the same bed or same room. This may be related to the increased transmission of viral infections that progress with wheezing in infancy, with or without bronchial hyper- reactivity.^13,28^ Elder et al. (1996)^13^ found an association between the presence of siblings in the household and wheezing during the first year of life. In our study, the presence of siblings in the household did not constitute a significant risk factor.

In a cross-sectional study, Rona et al. (1993)^29^ evaluated respiratory symptoms in 5,573 children from five to eleven years of age using an interview with the parents. The authors defined respiratory symptoms as the presence of asthma, cough and frequent wheezing. They reported that gestational age, but not birth weight, was associated with respiratory symptoms, principally wheezing. In our study, gestational age less than 28 weeks did not constitute a significant risk of obstructive syndrome. Some comments are in order on the work by Rona et al. (1993).^29^ These results were not statistically significant (CI: 0.85-1.51) and the data on the signs of obstructive syndrome were only obtained from observations by family members (gathered through a questionnaire), which may have led to information bias.

Smoking during pregnancy was associated with reduced pulmonary function, beginning in the first weeks of life and continuing for up to 18 months, and with the presence of wheezing, in an analysis of data obtained from a questionnaire among family members of 16,333 children aged six to seven years.^30^ In our study, 34% of the children were passively exposed to cigarette smoke. However, unlike the results of Elder et al. (1996),^13^ the fact that the parents smoked did not appear as a risk factor for either respiratory morbidity or the presence of wheezing.

Jaimes et al. (2003)^31^ reported a 1.8 times greater risk of lower respiratory infection among children who shared the same bed, although the difference was not statistically significant, since the confidence interval ranged from 1.0 to 3.7.

We did not find an increased risk of pneumonia for either the presence of other children under five in the household or smoking by the parents (mother or father). Similar results were described by Victora et al. (1994)^32^ and Jaimes et al. (2003),^31^ who also found no association between pneumonia and the presence of other children in the household or exposure to maternal smoking. According to these authors, the latter finding contrasts with previous studies, and one of the possibilities for this negative result is that exposure to parents' cigarette smoke may be associated with other forms of respiratory infection (bronchitis or bronchiolitis), and not with pneumonia.

In the bivariate analysis we found an association between extremely low birth weight and pneumonia. Low birth weight may contribute towards the occurrence of pneumonia due to the decreased immune response and compromised pulmonary function, because of the reduced diameter of the larger airways or obstruction of peripheral airways.^32^

Palta et al. (1998)^26^ reported that oxygen use at 30 days of life presented an adjusted odds ratio (OR) of 2.8 (CI: 1.4-5.8) for hospitalization due to respiratory disease. These authors reported that European authors such as Hakulinen et al. (1988)^33^ and Australian authors like Kitchen et al. (1990)^34^ did not show an association between neonatal factors and hospitalization. This was ascribed to differences in the care provided under the different health systems. In the bivariate analysis, we found a statistically significant association between the following neonatal variables and hospitalization: utilization of assisted ventilation, birth weight less than 1,000 g, apnea, neonatal pneumonia, and prolonged use of oxygen. The RR for prolonged use of oxygen was 2.03 (CI: 1.05-3.90). In a study by Cunningham et al. (1991),^35^ bronchopulmonary dysplasia presented an OR of 2.45 for hospitalization during the first two years of life. Our results for risk due to prolonged use of oxygen in relation to hospitalization during the first year of life agree with the results from the authors cited above.^26,35^

The need for mechanical ventilation increases the risk of lung injuries resulting from free oxygen radicals, and the exposure to high volume and inspiratory pressure that may cause barotrauma and respiratory epithelium injuries. Studies on animal models have already shown that mechanical ventilation and exposure to high oxygen concentrations can interfere with the normal postnatal alveolarization, thus causing a series of histopathological abnormalities.^24^ Pulmonary structure abnormalities can lead to higher incidence of future respiratory morbidity.^36^ Giffin et al. (1994)^22^ showed an association between mechanical ventilation and respiratory disease during the first three years of life. Our study also showed that oxygen use for 10 days or longer and mechanical ventilation were associated with respiratory morbidity during the first year of life.

According to our bivariate analysis, numerous factors were associated with respiratory morbidity. However, in the multivariate analysis, the covariables selected according to statistical significance that best explained respiratory morbidity were the following: neonatal pneumonia (RR: 5.15; CI: 1.34-19.87) and mechanical ventilation (RR: 3.29; CI: 1.33- 8.16), which is in agreement with the results from Giffin et al. (1994).^22^ Because of the need to include variables with major biological significance, we attempted a modeling process aimed at including such variables (abnormal pulmonary compliance and CT with three or more abnormalities). However these did not attain statistical significance. Thus, models C, D, and E (Table 7) evaluated the effect of each of these variables alone on model B. From comparing the p-values for each of the "new" variables introduced into each model with those for the "original" variables selected in model B (neonatal pneumonia and assisted ventilation), it could been seen that the "new" variables continued to have p-values lower than 0.3. They hardly modified the effect of the "original" variables that retained significance. However, in model E, the "new" variable introduced (abnormal pulmonary compliance and abnormal CT) presented borderline significance (p = 0.14), despite having an adjusted relative risk of close to three for respiratory morbidity. It also reduced the significance of the "original" variables in model B, thus also making them borderline. This effect may indicate a need to increase the sample size in order to reach statistical significance. It corroborates the existence of interaction between the variables "abnormal CT" and "abnormal compliance" and of effective control relating to confounding factors for this interaction, among the "original" variables in model B.

Identification of children at higher risk of respiratory disease during the first year of life allows for planning of approaches, both at the time of discharge and during follow-up. The children at greatest risk, i.e. those with a history of neonatal pneumonia, mechanical ventilation or abnormalities in pulmonary compliance or pulmonary structure could thus receive under greater surveillance than the others. This would be particularly so when viral diseases that worsened the obstructive symptoms were present.

## CONCLUSIONS

The mean values for pulmonary compliance and resistance in the neonates studied here are similar to those described in the literature.

We found a high rate of compromised pulmonary function just prior to hospital discharge. This was translated as abnormal pulmonary compliance in 40% of the children and abnormal pulmonary resistance in 59% of the cohort.

We found a high percentage (72%) of abnormal thoracic tomography.

More than 50% of our study sample presented respiratory morbidity over the course of the first year of life (corrected for prematurity).

The statistically significant risk factors for respiratory morbidity during the first year of life were neonatal pneumonia and use of assisted ventilation.

The results from the present study may contribute towards early identification of children at increased risk of respiratory disease during their first year of life.
